# Efficacy and safety of selective TYK2 inhibitor, deucravacitinib, in a phase II trial in psoriatic arthritis

**DOI:** 10.1136/annrheumdis-2021-221664

**Published:** 2022-03-03

**Authors:** Philip J Mease, Atul A Deodhar, Désirée van der Heijde, Frank Behrens, Alan J Kivitz, Jeffrey Neal, Jonghyeon Kim, Shalabh Singhal, Miroslawa Nowak, Subhashis Banerjee

**Affiliations:** 1 Swedish Medical Center/Providence St. Joseph Health and University of Washington, Seattle, Washington, USA; 2 Oregon Health & Science University, Portland, Oregon, USA; 3 Leiden University Medical Center, Leiden, The Netherlands; 4 Rheumatology and Fraunhofer Institute, Translational Medicine and Pharmacology (ITMP) & Fraunhofer Cluster of Excellence Immune-Mediated Diseases (CIMD), Goethe University, Frankfurt, Germany; 5 Department of Rheumatology, Altoona Center for Clinical Research, Duncansville, Pennsylvania, USA; 6 Arthritis Center of Lexington, University of Kentucky School of Medicine, Lexington, Kentucky, USA; 7 Bristol Myers Squibb, Princeton, New Jersey, USA

**Keywords:** arthritis, psoriatic, inflammation, therapeutics

## Abstract

**Objective:**

To evaluate the efficacy and safety of an oral selective tyrosine kinase 2 (TYK2) inhibitor, deucravacitinib, in patients with active psoriatic arthritis (PsA).

**Methods:**

In this double-blind, phase II trial, 203 patients with PsA were randomised 1:1:1 to placebo, deucravacitinib 6 mg once a day or 12 mg once a day. The primary endpoint was American College of Rheumatology-20 (ACR-20) response at week 16.

**Results:**

ACR-20 response was significantly higher with deucravacitinib 6 mg once a day (52.9%, p=0.0134) and 12 mg once a day (62.7%, p*=*0.0004) versus placebo (31.8%) at week 16. Both deucravacitinib doses resulted in significant improvements versus placebo (p≤0.05) in the multiplicity-controlled secondary endpoints of change from baseline in Health Assessment Questionnaire-Disability Index and Short Form-36 Physical Component Summary score and in Psoriasis Area and Severity Index-75 response. Improvements were also seen in multiple exploratory endpoints with deucravacitinib treatment. The most common adverse events (AEs) (≥5%) in deucravacitinib-treated patients were nasopharyngitis, upper respiratory tract infection, sinusitis, bronchitis, rash, headache and diarrhoea. There were no serious AEs and no occurrence of herpes zoster, opportunistic infections and major adverse cardiovascular events, or differences versus placebo in mean changes in laboratory parameters with deucravacitinib treatment.

**Conclusions:**

Treatment with the selective TYK2 inhibitor deucravacitinib was well tolerated and resulted in greater improvements than placebo in ACR-20, multiplicity-controlled secondary endpoints and other exploratory efficacy measures in patients with PsA. Larger trials over longer periods of time with deucravacitinib are warranted to confirm its safety profile and benefits in PsA.

**Trial registration number:**

NCT03881059.

**Key messages**

**What is already known about this subject?**

- Interleukin 23 is a key cytokine in the pathogenesis of psoriatic arthritis, psoriasis and other immune-mediated diseases, and its signalling is mediated by the intracellular kinase, tyrosine kinase 2 (TYK2).
- Deucravacitinib is a novel oral selective TYK2 inhibitor that binds to the unique regulatory domain of TYK2 with high selectivity, in contrast to inhibitors of closely related Janus kinases 1/2/3 that bind the conserved active domain.

**What does this study add?**

- Deucravacitinib at 6 mg and 12 mg doses once a day demonstrated greater efficacy versus placebo at week 16, with improvements observed across all American College of Rheumatology domains, enthesitis endpoints, and multiple patient-reported, psoriasis-related and composite outcomes in patients with active psoriatic arthritis.
- Treatment with deucravacitinib was generally well tolerated, and the safety and laboratory parameter profile of deucravacitinib was consistent with its selective mechanism of action and with that observed in an earlier phase II psoriasis trial and recently reported phase III trials in psoriasis.

**How might this impact on clinical practice or future developments?**

- The options for targeted oral therapies in psoriatic arthritis are limited; deucravacitinib, which demonstrated improved efficacy versus placebo and was well tolerated, may be a promising option for treatment of patients with active psoriatic arthritis.

**Video abstract:** Disclaimer: this video summarises a scientific article published by BMJ Publishing Group Limited (BMJ). The content of this video has not been peer-reviewed and does not constitute medical advice. Any opinions expressed are solely those of the contributors. Viewers should be aware that professionals in the field may have different opinions. BMJ does not endorse any opinions expressed or recommendations discussed. Viewers should not use the content of the video as the basis for any medical treatment. BMJ disclaims all liability and responsibility arising from any reliance placed on the content.

## Introduction

Psoriatic arthritis (PsA) is a heterogeneous disease with diverse manifestations, including arthritis, enthesitis, dactylitis, and skin and nail lesions.^1 2^ Up to 30% of patients with psoriasis (PsO) can develop PsA.^1 2^ Patients with PsA are at an increased risk of developing serious comorbidities,^2 3^ which can increase the risk of death.^4^ A substantial proportion of patients with PsA are inadequately treated with currently available therapeutic options; many of these medications have safety concerns and have inconvenient dosing, and few patients reach treatment targets, such as achievement of minimal disease activity (MDA). This results in disease progression and disability, frequent medication switching, and higher overall treatment costs.^5 6^ Therapies with new modes of action that are safe, effective and have convenient dosing are needed to control the spectrum of disease manifestations and improve the quality of life of patients with PsA as another option for treatment, including in those who do not respond to other modalities.^7 8^

Tyrosine kinase 2 (TYK2) is an intracellular kinase that is a member of the Janus kinase (JAK) family of kinases which signal through the JAK–signal transducer and activator of transcription pathway. TYK2 mediates signalling by cytokines such as interleukin (IL) 23 that are involved in the pathogenesis of PsO, PsA and other immune-mediated diseases.^9 10^ TYK2 signalling pathways are restricted to select immune pathways unlike those of the other members of the JAK family, JAK 1/2/3, which are involved in broader immune (eg, T cells and natural killer cells) as well as in extraimmune pathways (eg, bone marrow effects, lipid metabolism).^11^ IL-23 is involved in the activation and proliferation of Th17 cells linked to sustained inflammatory responses in the skin and joints in PsA, and anti-IL-23 antibodies have shown efficacy in PsO and PsA.^2 12^ Patients with early PsA who do not achieve MDA with standard methotrexate therapy have higher levels of IL-23 than those who respond to methotrexate.^13^

Deucravacitinib is a novel oral selective TYK2 inhibitor with a unique mechanism of action distinct from that of inhibitors of JAK 1/2/3.^9^ Deucravacitinib binds to the regulatory or pseudokinase domain of TYK2 and inhibits the enzyme via a conformational change that locks the enzyme in an inactive state. This is in contrast to inhibitors of JAK 1/2/3 and other kinases that act on the conserved active domains at the adenosine 5′-triphosphate binding site. This allosteric inhibition results in 100-fold to 2000-fold selectivity for TYK2 over JAK 1/2/3 in in vitro cellular assays.^9^

Deucravacitinib was shown to be efficacious in phase II and phase III trials in PsO and was well tolerated overall with generally mild to moderate adverse events (AEs).^14 15^ No opportunistic infections or laboratory abnormalities characteristic of JAK 1/2/3 inhibitors were observed with deucravacitinib treatment.^14–18^

This phase II trial evaluated the efficacy and safety of deucravacitinib in patients with active PsA at two doses. Deucravacitinib was administered at randomisation at a dosage of 6 mg once a day, the dosage that was selected for phase III trials in PsO based on the phase II results, as well as at a dosage of 12 mg once a day to evaluate whether higher exposures could lead to better efficacy in joints, as has been seen with some other agents.^14 15 19 20^

## Methods

### Trial design

This randomised, multicentre, double-blind, phase II trial was conducted in the Czech Republic, Germany, Hungary, Poland, Spain, Russia and USA. The results from the initial 16-week placebo-controlled period (part A) of the trial (see study design in online supplemental figure S1) are presented in this article. Eligible patients had a diagnosis of PsA for ≥6 months and fulfilled the Classification Criteria for Psoriatic Arthritis (CASPAR) at screening, had active joint disease (at least three tender and at least three swollen joints), a high-sensitivity C reactive protein (hs-CRP) level of ≥3 mg/L (upper limit of normal, 5 mg/L) and ≥1 plaque PsO lesion (≥2 cm).^21^ They had to have failed to respond or were intolerant to ≥1 prior therapy, which could include non-steroidal anti-inflammatory drugs, corticosteroids, conventional synthetic disease-modifying antirheumatic drugs (csDMARD) and/or one tumour necrosis factor inhibitor (TNFi). Concomitant use of a csDMARD (eg, methotrexate, leflunomide, sulfasalazine or hydroxychloroquine) was permitted if used for ≥3 months with a stable dose for ≥28 days prior to the trial. Additional eligibility criteria are listed in the online supplemental materials.

10.1136/annrheumdis-2021-221664.supp1 Supplementary data

10.1136/annrheumdis-2021-221664.supp2 Supplementary data

Eligible patients were randomised 1:1:1 to oral placebo once a day, deucravacitinib 6 mg once a day or deucravacitinib 12 mg once a day for 16 weeks. Randomisation was stratified according to previous TNFi use (experienced/naïve) and body weight (≥90 kg and <90 kg). A randomisation list was generated by an interactive response technology using a permuted block design within each combination of stratum level. Investigative site staff, study sponsor and patients remained blinded to treatment assignment. Patients provided written informed consent before trial entry.

### Endpoints

The primary endpoint was American College of Rheumatology-20 (ACR-20) response at week 16, defined as meeting the following criteria: ≥20% improvement from baseline in the number of tender joints (68 total joint count); ≥20% improvement from baseline in the number of swollen joints (66 total joint count); and ≥20% improvement from baseline in at least three of the following five domains: patient global assessment of pain, patient global assessment of disease activity, physician global assessment of disease activity, Health Assessment Questionnaire-Disability Index (HAQ-DI) and hs-CRP. Multiplicity-controlled secondary efficacy endpoints were evaluated using hierarchical testing at week 16 and included (1) improvement from baseline in physical function as measured by HAQ-DI; (2) improvement in psoriatic skin lesions as measured by Psoriasis Area and Severity Index (PASI) 75 response (≥75% reduction from baseline in PASI scores) in patients with ≥3% body surface area involvement at baseline; and (3) change from baseline in the quality of life measure, Short Form-36 (SF-36) Physical Component Summary (PCS) score. Additional endpoints evaluated at week 16 which were not multiplicity-controlled included the proportion of patients achieving higher ACR thresholds of efficacy (ACR-50 and ACR-70 responses); HAQ-DI response (≥0.35 improvement from baseline (minimum clinically important difference in PsA)); resolution of enthesitis (Leeds Enthesitis Index (LEI) of 0 in patients with LEI ≥1 at baseline); resolution of dactylitis (score of 0 in patients with ≥1 tender and swollen digit at baseline); mean changes from baseline in Psoriatic Arthritis Disease Activity Score (PASDAS), Disease Activity Index for Psoriatic Arthritis (DAPSA) and SF-36 Mental Component Summary (MCS) score; and achievement of MDA (defined as achieving at least five of the following: tender joint count ≤1; swollen joint count ≤1; PASI ≤1 or body surface area ≤3%; tender entheseal points ≤1; patient global assessment of pain ≤15; patient global assessment of disease activity ≤20; and HAQ-DI ≤0.5). A full listing of all endpoints is provided in the online supplemental materials. Comparisons between treatment groups over time were also evaluated as exploratory endpoints. Safety assessments, including reporting of AEs, physical examinations, vital signs, ECG and laboratory parameters were conducted periodically throughout the trial.

### Statistical analysis

Sample size and power determination are described in the online supplemental materials. The primary efficacy analysis used a logistic regression model to assess whether there was a dose–response trend between ACR-20 response and dose level at week 16. This model included dose level as a continuous variable, and TNFi use (experienced/naïve) and body weight (≥90 kg/<90 kg) as covariates. The OR versus placebo and the corresponding two-sided 95% CI were estimated by Cochran-Mantel-Haenszel test with stratification factors (body weight and TNFi use). Patients who discontinued the trial early, started a prohibited treatment, were lost to follow-up or had no ACR-20 assessments at week 16 had outcomes imputed as non-responses in an intention-to-treat analysis. A Cochran-Mantel-Haenszel test was applied to assess the robustness of the results for the primary endpoint by predefined subgroups based on stratification factors.

Statistical analysis of secondary endpoints at week 16 was performed in the following hierarchical order to control for multiplicity: (1) change from baseline in HAQ-DI score, (2) PASI-75 response and (3) change from baseline in SF-36 PCS. Secondary endpoint analyses are further described in the online supplemental materials. Any reported p values in the tests for additional endpoints will be considered nominal.

### Patient and public involvement

Patients and the public were not involved in the design, conduct, reporting or dissemination of this research.

## Results

### Patients

The trial was initiated on 28 March 2019, with the last patient’s last visit of the 16-week placebo-controlled period occurring on 27 April 2020. Of 314 patients screened, 203 were randomised and received treatment (placebo, n=66; deucravacitinib 6 mg once a day, n=70; deucravacitinib 12 mg once a day, n=67). Of the randomised patients, 180 (89%) completed 16 weeks of treatment, with the most common causes of discontinuation being AEs and patient withdrawal across the treatment arms (online supplemental figure S2).

10.1136/annrheumdis-2021-221664.supp3 Supplementary data

Demographic and baseline disease characteristics were overall similar across the three treatment groups. The mean age was 49.8 years, 51.2% were female, 98% were Caucasians, the mean body weight was 88.6 kg, 65.0% were being treated with csDMARDs at baseline and 15.8% had previously been treated with a TNFi (table 1). In addition, the median PsA duration (from diagnosis) was 4.5 years, the mean swollen joint count was 11.3, the mean tender joint count was 18.1, enthesitis (LEI) was present in 47.3%, dactylitis in 38.9%, and the mean PASI score was 8.5 in those with body surface area of involvement ≥3%.

**Table 1:** Baseline demographic and clinical characteristics

	Total N=203	Placebo n=66	Deucravacitinib	
			6 mg once a day n=70	12 mg once a day n=67
Demographics				
Age, years, mean (SD)	49.8 (13.5)	48.5 (13.2)	50.5 (13.7)	50.5 (13.8)
Female, n (%)	104 (51.2)	40 (60.6)	30 (42.9)	34 (50.7)
White, n (%)	199 (98.0)	65 (98.5)	67 (95.7)	67 (100.0)
Body weight, kg, mean (SD)	88.6 (19.0)	90.5 (22.7)	86.4 (16.6)	89.1 (17.3)
<90 kg, n (%)	104 (51.2)	33 (50.0)	36 (51.4)	35 (52.2)
≥90 kg, n (%)	99 (48.8)	33 (50.0)	34 (48.6)	32 (47.8)
BMI, kg/m^2^, mean (SD)	30.4 (6.0)	31.2 (7.2)	29.6 (5.4)	30.3 (5.4)
Prior/concomitant medications				
Use of csDMARD, n (%)	132 (65.0)	44 (66.7)	45 (64.3)	43 (64.2)
Use of methotrexate, n (%)	111 (54.7)	39 (59.1)	35 (50.0)	37 (55.2)
Weekly dose, mg, mean (SD)	16.5 (4.7)	16.7 (4.8)	16.4 (4.9)	16.5 (4.6)
Prior TNFi use, n (%)				
1	31 (15.3)	11 (16.7)	12 (17.1)	8 (11.9)
2	1 (0.5)	0	0	1 (1.5)
Oral steroid use, n (%)	25 (12.3)	12 (18.2)	7 (10.0)	6 (9.0)
Daily dose, mg, mean (SD)	4.0 (1.7)	4.4 (1.9)	3.7 (1.3)	3.5 (1.6)
Disease parameters				
Psoriatic arthritis disease duration from diagnosis, years, median (range)	4.5 (0.1–42.8)	4.5 (0.6–22.9)	5.3 (0.1–42.8)	3.8 (0.6–27.7)
Tender joint count, mean (SD)	18.1 (10.7)	16.9 (9.8)	18.1 (10.3)	19.4 (11.8)
Swollen joint count, mean (SD)	11.3 (7.9)	10.5 (7.7)	11.9 (7.0)	11.3 (9.0)
Pain in mm, VAS, mean (SD)*	64.1 (18.7)	64.9 (18.2)	63.6 (21.7)	63.8 (15.9)
HAQ-DI, mean (SD)	1.3 (0.6)	1.3 (0.6)	1.3 (0.6)	1.3 (0.6)
hs-CRP, mg/L, mean (SD)	18.2 (29.0)	20.4 (39.1)	17.6 (23.6)	16.5 (21.7)
Psoriasis with ≥3% BSA, n (%)	165 (81.3)	54 (81.8)	59 (84.3)	52 (77.6)
PASI-75 score in patients with ≥3% BSA				
Mean (SD)	8.5 (6.7)	9.1 (7.4)	8.5 (6.8)	7.9 (5.9)
Range	1.2–33.8	1.2–31.4	1.6–33.8	1.4–31.8
Enthesitis, Leeds Index ≥1, n (%)	96 (47.3)	31 (47.0)	39 (55.7)	26 (38.8)
Leeds Index in those with enthesitis, mean (SD)	2.7 (1.6)	2.8 (1.7)	2.5 (1.6)	2.9 (1.4)
Dactylitis, n (%)	79 (38.9)	25 (37.9)	30 (42.6)	24 (35.8)

*VAS scale ranges from 0–100 mm, with higher values indicating worse pain.

BMI, body mass index; BSA, body surface area; csDMARD, conventional synthetic disease-modifying antirheumatic drug; HAQ-DI, Health Assessment Questionnaire-Disability Index; hs-CRP, high-sensitivity C reactive protein; PASI-75, 75% improvement from baseline in Psoriasis Area and Severity Index; TNFi, tumour necrosis factor inhibitor; VAS, Visual Analogue Scale.

### Efficacy

**Figure 1:**
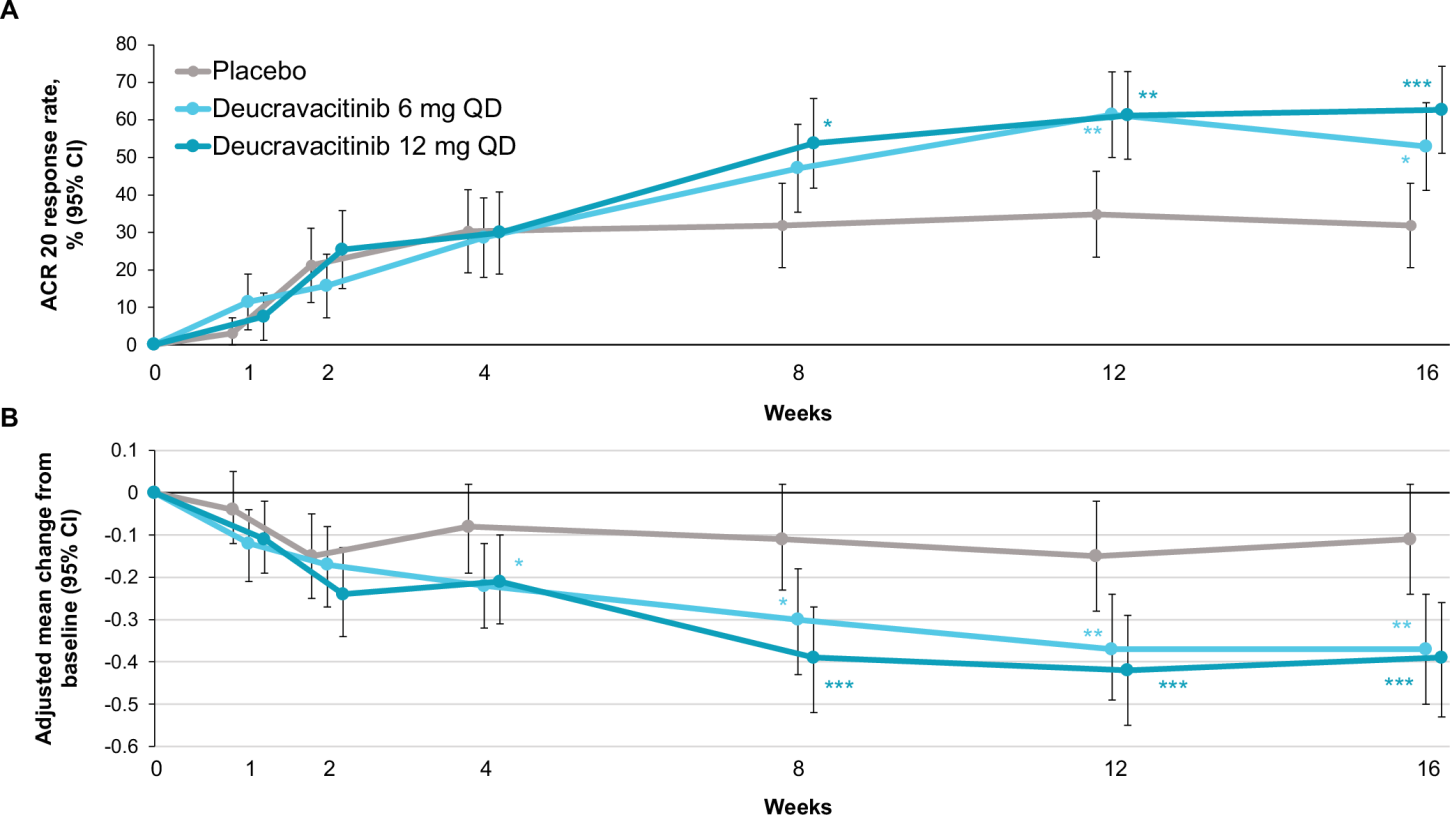
ACR-20 response and change in HAQ-DI score over time. Supporting values are shown in online supplemental table S4. (A) Time course of ACR-20 response through week 16. Response rates are reported in the intention-to-treat population (ie, all randomised patients) with non-responder imputation; patients who discontinued the trial early, started a prohibited treatment, were lost to follow-up or had no ACR assessments had outcomes imputed as non-responses. (B) Adjusted mean change from baseline in HAQ-DI score through week 16. Placebo, n=66; deucravacitinib 6 mg once a day, n=70; deucravacitinib 12 mg once a day, n=67. P values indicate a difference from placebo: *p<0.05, **p≤0.01, ***p≤0.001, adjusted for multiplicity at week 16 only. ACR, American College of Rheumatology; HAQ-DI, Health Assessment Questionnaire-Disability Index; QD, once a day.

The study met its primary objective, with ACR-20 response being significantly higher with deucravacitinib 6 mg once a day (52.9%) and 12 mg once a day (62.7%) versus placebo (31.8%) at week 16. The adjusted OR (95% CI) for deucravacitinib 6 mg once a day versus placebo was 2.4 (1.2 to 4.8) (p=0.0134) and for deucravacitinib 12 mg once a day versus placebo was 3.6 (1.8 to 7.4) (p=0.0004). Numerical improvements in ACR-20 response were observed from week 8 onwards at both deucravacitinib doses versus placebo (figure 1A). Higher ACR-20 response was seen with deucravacitinib treatment versus placebo regardless of prior TNFi exposure (experienced vs naïve), body weight (<90 kg vs ≥90 kg) or gender (male vs female) (online supplemental figure S3). Mean improvements in individual ACR components from baseline were greater with each deucravacitinib dose versus placebo (online supplemental table S1).

10.1136/annrheumdis-2021-221664.supp4 Supplementary data

10.1136/annrheumdis-2021-221664.supp5 Supplementary data

10.1136/annrheumdis-2021-221664.supp8 Supplementary data

Other efficacy endpoints at week 16 were also numerically higher with both deucravacitinib doses compared with placebo, including ACR-50, ACR-70 and HAQ-DI responses (nominal p≤0.05; table 2). The mean improvements from baseline in HAQ-DI scores at week 16 were significantly higher with deucravacitinib 6 mg and 12 mg once a day versus placebo (p≤0.002), with improvements evident as early as week 4 with both deucravacitinib doses (figure 1B). Higher PASI-75 response was observed in patients with PsO involving ≥3% body surface area at baseline with deucravacitinib 6 mg once a day (42.4%; adjusted OR 2.9 (95% CI 1.3 to 6.7); p=0.0136) and 12 mg once a day (59.6%; OR 5.8 (95% CI 2.4 to 13.8); p<0.0001) versus placebo (20.4%) at week 16. Significantly greater improvements from baseline were seen at week 16 with deucravacitinib treatment at both doses versus placebo in SF-36 PCS, as well as numerical improvements in SF-36 MCS scores (p≤0.0062 and nominal p≤0.0263, respectively; table 2). Higher numbers of patients treated with deucravacitinib 6 mg once a day and 12 mg once a day versus placebo achieved enthesitis resolution (51.3%, 50.0%, 22.6%), dactylitis resolution (76.7%, 79.2%, 60.0%) and MDA (22.9%, 23.9%, 7.6%), and showed greater mean change from baseline in PASDAS (−2.0, –2.1, −1.1) and DAPSA scores (−23.2, –25.6, −13.3), respectively (table 2).

**Table 2:** Efficacy endpoints at week 16

Endpoint	Placebo n=66	Deucravacitinib	
		6 mg once a day n=70	12 mg once a day n=67
Primary endpoint			
ACR-20			
Response rate, % (95% CI)	31.8 (20.6 to 43.1)	52.9 (41.2 to 64.6)	62.7 (51.1 to 74.3)
Adjusted OR vs placebo (95% CI)		2.4 (1.2 to 4.8)	3.6 (1.8 to 7.4)
P value		0.0134*	0.0004*
Secondary endpoints			
HAQ-DI			
Adjusted mean change from baseline (95% CI)	−0.1 (−0.2 to 0.0)	−0.4 (−0.5 to −0.2)	−0.4 (−0.5 to −0.3)
Difference from placebo (95% CI)		−0.3 (−0.4 to −0.1)	−0.3 (−0.5 to −0.1)
P value		0.0020*	0.0008*
PASI-75			
Response rate, % (95% CI)	20.4 (9.6 to 31.1)	42.4 (29.8 to 55.0)	59.6 (46.3 to 73.0)
Adjusted OR vs placebo (95% CI)		2.9 (1.3 to 6.7)	5.8 (2.4 to 13.8)
P value		0.0136*	<0.0001*
SF-36 PCS			
Adjusted mean change from baseline (95% CI)	2.3 (0.4 to 4.2)	5.6 (3.8 to 7.5)	5.8 (3.9 to 7.7)
Difference from placebo (95% CI)		3.3 (0.9 to 5.7)	3.5 (1.1 to 5.9)
P value		0.0062*	0.0042*
Additional endpoints			
ACR-50			
Response rate, % (95% CI)	10.6 (3.2 to 18.0)	24.3 (14.2 to 34.3)	32.8 (21.6 to 44.1)
Adjusted OR vs placebo (95% CI)		2.7 (1.1 to 7.1)	4.2 (1.7 to 10.9)
P value		0.0326	0.0016
ACR-70			
Response rate, % (95% CI)	1.5 (0.0 to 4.5)	14.3 (6.1 to 22.5)	19.4 (9.9 to 28.9)
Adjusted OR vs placebo (95% CI)		12.0 (1.5 to 99.3)	19.0 (2.3 to 155.2)
P value		0.0044	0.0003
HAQ-DI			
Response rate†, % (95% CI)	15.2 (6.5 to 23.8)	38.6 (27.2 to 50.0)	40.3 (28.6 to 52.0)
Adjusted OR vs placebo (95% CI)		3.8 (1.6 to 8.8)	3.7 (1.6 to 8.4)
P value		0.0019	0.0015
SF-36 MCS			
Adjusted mean change from baseline (95% CI)	0.7 (−1.3 to 2.7)	3.6 (1.7 to 5.5)	3.5 (1.5 to 5.5)
Adjusted mean difference from placebo (95% CI)		2.9 (0.4 to 5.3)	2.8 (0.3 to 5.3)
P value		0.0211	0.0263
Enthesitis resolution (LEI)	n=31	n=39	n=26
Response rate, % (95% CI)	22.6 (7.9 to 37.3)	51.3 (35.6 to 67.0)	50.0 (30.8 to 69.2)
Adjusted OR vs placebo (95% CI)		3.6 (1.3 to 10.3)	3.4 (1.1 to 10.7)
P value		0.0138	0.0393
Dactylitis resolution	n=25	n=30	n=24
Response rate, % (95% CI)	60.0 (40.8 to 79.2)	76.7 (61.5 to 91.8)	79.2 (62.9 to 95.4)
Adjusted OR vs placebo (95% CI)		2.2 (0.7 to 7.1)	2.8 (0.8 to 10.5)
P value		NA	NA
PASDAS			
Adjusted mean change from baseline (95% CI)	−1.1 (−1.5 to −0.7)	−2.0 (−2.4 to −1.6)	−2.1 (−2.5 to −1.8)
Adjusted mean difference from placebo (95% CI)		−0.9 (−1.4 to −0.4)	−1.1 (−1.5 to −0.6)
P value		0.0003	<0.0001
DAPSA			
Adjusted mean change from baseline (95% CI)	−13.3 (−17.7 to −9.0)	−23.2 (−27.5 to −19.0)	−25.6 (−30.0 to −21.2)
Adjusted mean difference from placebo (95% CI)		−9.9 (−15.3 to −4.5)	−12.3 (−17.7 to −6.8)
P value		0.0004	<0.0001
MDA			
Response rate, % (95% CI)	7.6 (1.2 to 14.0)	22.9 (13.0 to 32.7)	23.9 (13.7 to 34.1)
OR vs placebo (95% CI)		3.8 (1.3 to 11.1)	4.1 (1.4 to 12.2)
P value		0.0119	0.0068

*Statistical analyses of primary and secondary endpoints at week 16 were adjusted for multiplicity. Additional endpoints were not controlled for multiple comparisons and nominal p values are reported.

†Response criteria of ≥0.35 improvement from baseline (minimum clinically important difference in PsA).

ACR, American College of Rheumatology; DAPSA, Disease Activity Index for Psoriatic Arthritis; HAQ-DI, Health Assessment Questionnaire-Disability Index; LEI, Leeds Enthesitis Index; MCS, Mental Component Summary; MDA, minimal disease activity; NA, not analysed; PASDAS, Psoriatic Arthritis Disease Activity Score; PASI, Psoriasis Area and Severity Index; PCS, Physical Component Summary; PsA, psoriatic arthritis; SF-36, Short Form-36.

### Safety

**Figure 2:**
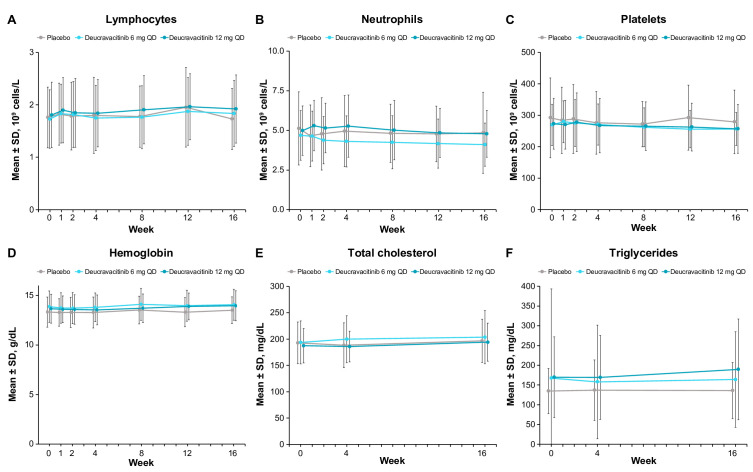
Laboratory parameters over 16 weeks (mean±SD): (A) lymphocytes, (B) neutrophils, (C) platelets, (D) haemoglobin, (E) total cholesterol and (F) triglycerides. Supporting values are shown in online supplemental table S5. QD, once a day.

AEs were observed at a higher frequency at both deucravacitinib doses (65.7%) compared with placebo (42.4%) (table 3). The most common AEs (≥5%) in deucravacitinib-treated patients were nasopharyngitis, upper respiratory tract infection, sinusitis, bronchitis, rash, diarrhoea and headache (table 3), with most AEs being of mild to moderate severity. Acne was reported in 2 of 70 (2.9%) patients in the 6 mg once a day deucravacitinib treatment group, 1 of 67 (1.5%) in the 12 mg once a day group, and 0 of 66 (0.0%) in the placebo group; dermatitis acneiform was reported in 2 of 70 (2.9%), 2 of 67 (3.0%) and 0 of 66 (0.0%), respectively. No serious AEs (including serious infections) were reported in deucravacitinib-treated patients. There were no thrombotic events in the deucravacitinib groups; one patient in the placebo group with a family history of thrombophilia had a serious AE of deep vein thrombosis. There was no occurrence of herpes zoster, tuberculosis, opportunistic infection or malignancy observed with deucravacitinib treatment at either dose. AEs that resulted in treatment discontinuation occurred in one patient in the placebo group (PsO), three patients in the deucravacitinib 6 mg once a day group (bronchitis, rash and rosacea) and four patients in the deucravacitinib 12 mg once a day group (furuncle, urticaria, mouth ulceration and multiple events in one patient: gastro-oesophageal reflux disease, nausea, dizziness, headache and increased blood pressure). No differences in mean change in laboratory parameters (haematology (lymphocyte, neutrophil, platelet and haemoglobin levels), serum lipids (total cholesterol and triglyceride levels) or chemistry (alanine aminotransferase, aspartate aminotransferase, creatine phosphokinase and creatinine)) were observed between deucravacitinib and placebo treatment arms across 16 weeks of treatment (figure 2 and online supplemental table S2). Majority of the patients had laboratory parameters within normal ranges (Common Terminology Criteria for Adverse Events grade 0) throughout the study; shifts to grades 3 or 4 from baseline, when treatment decisions would need to be made, were uncommon, with no clinically meaningful differences overall between the treatment arms (online supplemental table S3).

10.1136/annrheumdis-2021-221664.supp6 Supplementary data

10.1136/annrheumdis-2021-221664.supp7 Supplementary data

**Table 3:** Summary of safety

Adverse event (AE), n (%)	Placebo n=66	Deucravacitinib	
		6 mg once a day n=70	12 mg once a day n=67
Total AEs	28 (42.4)	46 (65.7)	44 (65.7)
Treatment-related AEs	6 (9.1)	22 (31.4)	17 (25.4)
Deaths	0	0	0
Serious AEs	1 (1.5)	0	0
Treatment discontinuation due to AEs	1 (1.5)	3 (4.3)	4 (6.0)
AEs occurring in ≥5% of patients in any treatment group			
Nasopharyngitis	5 (7.6)	4 (5.7)	12 (17.9)
Upper respiratory tract infection	0	4 (5.7)	1 (1.5)
Sinusitis	0	0	5 (7.5)
Bronchitis	1 (1.5)	4 (5.7)	0
Headache	3 (4.5)	5 (7.1)	1 (1.5)
Rash	0	3 (4.3)	4 (6.0)
Diarrhoea	0	4 (5.7)	0

Includes events with a start date between the first dose and the week 16 visit date (inclusive), or between the first dose and 30 days after the last dose of study drug for patients who discontinued early.

10.1136/annrheumdis-2021-221664.supp9 Supplementary data

## Discussion

Deucravacitinib is an oral selective TYK2 inhibitor that targets the unique pseudokinase domain of the enzyme and inhibits TYK2-mediated pathways with high selectivity over other JAKs (JAK 1/2/3).^9^ In this relatively small, phase II study, deucravacitinib given at two doses, 6 mg once a day and 12 mg once a day, showed higher responses than placebo in multiple domains of PsA, including arthritis, enthesitis, dactylitis and skin inflammation. Although a decrease in clinical efficacy in women compared with men has been observed in some other PsA trials,^22^ a diminution in ACR-20 responses with deucravacitinib treatment in women versus men was not seen in this trial. In addition, significant improvements were observed in several patient-reported outcome measures, including physical function (HAQ-DI) and the quality of life measure SF-36 PCS, with deucravacitinib treatment. Differences from placebo were noted as early as week 4 for patient-reported outcomes and week 8 for ACR responses. Higher responses were also seen with deucravacitinib treatment versus placebo in SF-36 MCS and the composite measures of disease activity, PASDAS and DAPSA. The composite measure of low disease activity, MDA, is a treat-to-target goal in the treatment of PsA and reflects meaningful benefits across multiple disease domains in PsA.^23^ A substantial proportion of patients (approximately 23%) were able to achieve MDA with deucravacitinib treatment versus placebo (7.6%) by week 16. The beneficial effects with deucravacitinib treatment overall did not appear to be dose-dependent, as comparable responses were observed in the two groups across a majority of endpoints (eg, LEI, HAQ-DI responders, SF-36 PCS and MCS change from baseline, PASDAS improvements from baseline, MDA). However, few endpoints, including PASI-75, ACR-50 and ACR-70, did exhibit numerical differences between dose groups at week 16.

Deucravacitinib was generally well tolerated in patients with PsA, and the safety profile was consistent with that previously described earlier in PsO studies.^14 15^ AEs resulting in treatment discontinuation were few and were not specific to any organ system. The most common AE category was infections of the upper respiratory tract, which did not require treatment in the majority of cases and none led to discontinuation; this is consistent with the mechanism of action of deucravacitinib. Skin events of interest observed in the phase II PsO trial,^14^ including acne and dermatitis acneiform, occurred more frequently in patients treated with deucravacitinib than with placebo in this phase II PsA trial; however, neither occurred in more than 3.0% of the patients in any deucravacitinib treatment arm in the current study. No cases of herpes zoster infection, tuberculosis, opportunistic infections, malignancies or thromboembolic events were observed in deucravacitinib-treated patients. Changes in laboratory measures that are commonly observed with inhibitors of JAK 1/2/3 and are clinically meaningful, such as in haematological parameters, lipid levels and chemistry parameters, were not observed with deucravacitinib treatment, demonstrating the selectivity for TYK2 versus JAK 1/2/3.^10^

The study has some limitations. The sample size was relatively small and the results are reported over only 16 weeks of treatment, which limit the generalisability of our findings.

In conclusion, selective inhibition of TYK2 with deucravacitinib is a promising therapeutic option for PsA. Deucravacitinib showed efficacy across multiple disease domains and patient-reported outcomes and has a safety profile that is consistent with its mechanism of action and with that observed in previous phase II and phase III trials in PsO.^14 15^ Larger trials over longer durations are warranted to establish the long-term efficacy and safety profile of deucravacitinib in patients with active PsA.

## Previous publication

1. ACR Convergence (2020) American College of Rheumatology 2020 Annual Scientific Meeting. Mease PJ, *et al*. Efficacy and Safety of Deucravacitinib (BMS-986165), an Oral, Selective Tyrosine Kinase 2 Inhibitor, in Patients With Active Psoriatic Arthritis: Results From a Phase 2, Randomized, Double-Blind, Placebo-Controlled Trial. Poster presentation: November 9, 2020.
2. EADV (2021) European Academy of Dermatology and Venereology – 30th Congress (Virtual). Mease PJ, *et al*. Efficacy of Deucravacitinib, an Oral, Selective Tyrosine Kinase 2 Inhibitor, in Musculoskeletal Manifestations of Active Psoriatic Arthritis in a Phase 2, Randomized, Double-Blind, Placebo-Controlled Trial. Poster presentation: September 29, 2021.
3. CRA (2021) Chinese Rheumatology Association - 25th National Academic College of Rheumatology Conference. Mease P, *et al*. Efficacy and Safety of Deucravacitinib (BMS-986165), an Oral, Selective Tyrosine Kinase 2 Inhibitor, in Patients With Active Psoriatic Arthritis: Results From a Phase 2, Double-Blind, Randomized, Placebo-Controlled Trial. Poster presentation: May 20, 2021.
4. EULAR (2021) European Alliance of Associations for Rheumatology - EULAR 2021. Mease PJ, *et al*. Efficacy of Deucravacitinib, an Oral, Selective Tyrosine Kinase 2 Inhibitor, in Musculoskeletal Manifestations of Active Psoriatic Arthritis in a Phase 2, Randomized, Double-Blind, Placebo-Controlled Trial. Oral presentation: June 4, 2021.
5. EULAR (2021) European Alliance of Associations for Rheumatology - EULAR 2021. Mease PJ, *et al*. Efficacy and Safety of Deucravacitinib, an Oral, Selective Tyrosine Kinase 2 Inhibitor, in Patients With Active Psoriatic Arthritis: Results From a Phase 2, Randomized, Double-Blind, Placebo-Controlled Trial. Poster presentation: June 4, 2021.
6. IFPA (2021) International Federation of Psoriasis Associations - 6th World Psoriasis & Psoriatic Arthritis Conference. Mease PJ, *et al*. Efficacy and Safety of Deucravacitinib, an Oral, Selective Tyrosine Kinase 2 Inhibitor, in Patients With Active Psoriatic Arthritis: Results From a Phase 2, Randomized, Double-Blind, Placebo-Controlled Trial. Poster presentation: June 30, 2021.
7. JSPR (2021) Japanese Society for Psoriasis Research - 36th Annual Meeting. Habiro K, Mease PJ, *et al*. Efficacy and Safety of TYK2 Inhibitor Deucravacitinib in Patients With Active Psoriatic Arthritis: Global Phase 2 Clinical Study. Oral presentation: September 3, 2021.
8. DGRh (2021) Deutschen Gesellschaft für Rheumatologie - 49 Kongress. Mease PJ, *et al*. Efficacy and Safety of Deucravacitinib, an Oral, Selective Tyrosine Kinase 2 Inhibitor, in Patients With Active Psoriatic Arthritis: Results From a Phase 2, Randomized, Double-Blind, Placebo-Controlled Trial. Poster presentation: September 15, 2021.

## Data Availability

Data are available upon reasonable request. Bristol Myers Squibb’s policy on data sharing is found at https://www.bms.com/researchers-and-partners/independent-research/data-sharing-request-process.html
