# Supplementary material for: Efficacy and safety of selective TYK2 inhibitor, deucravacitinib, in a phase II trial in psoriatic arthritis
Source: Ann Rheum Dis. 2022 Mar 3;81(6):815–22. doi: 10.1136/annrheumdis-2021-221664 (PMC9120409; doi:10.1136/annrheumdis-2021-221664)
Supplement: Supplementary data [file annrheumdis-2021-221664supp001.pdf]

**Supplemental Figure S1. Study Design**

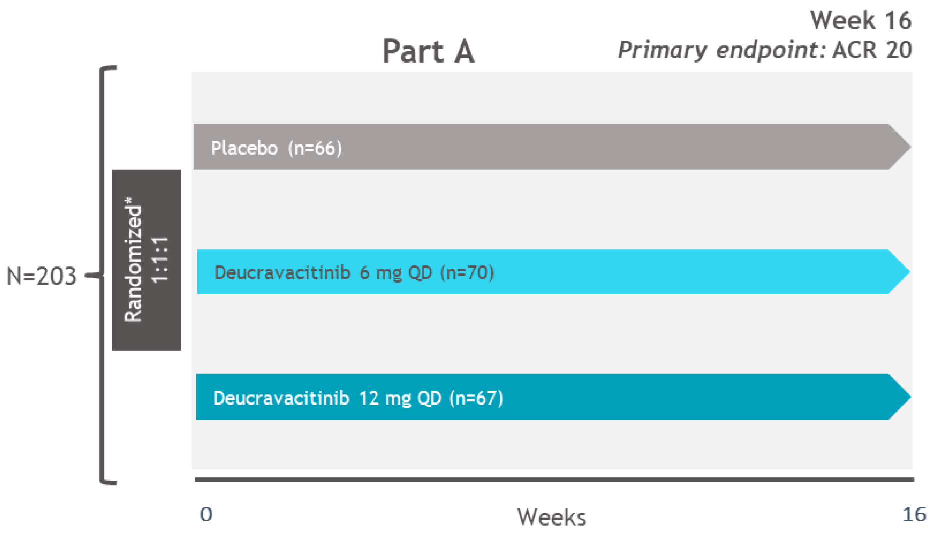


*Patients who completed the 16 weeks of double-blind treatment (Part A) were eligible for participation in an optional blinded long-term 36-week extension trial with active treatment (Part B).

ACR, American College of Rheumatology; QD, once daily.
