# Supplementary material for: Efficacy and safety of selective TYK2 inhibitor, deucravacitinib, in a phase II trial in psoriatic arthritis
Source: Ann Rheum Dis. 2022 Mar 3;81(6):815–22. doi: 10.1136/annrheumdis-2021-221664 (PMC9120409; doi:10.1136/annrheumdis-2021-221664)
Supplement: Supplementary data [file annrheumdis-2021-221664supp002.pdf]

Supplemental Material

METHODS

Key exclusion criteria

Patients were excluded if they had received any JAK inhibitors in the past, or had been treated with systemic non-biologic treatments for psoriasis and/or systemic immunosuppressant treatment within 4 weeks prior to the trial, or had used topical or shampoo treatments that could affect psoriasis evaluation within 2 weeks of the trial.

Treatment with targeted synthetic DMARDs (eg, apremilast) was not permitted during the study, but patients previously treated with apremilast were eligible if they discontinued treatment ≥4 weeks prior to trial start.

Patients were excluded if they had nonplaque psoriasis or another autoimmune condition, had tuberculosis, or had a history or evidence of active infections within 7 days prior to the start of the study.

Endpoints

Primary endpoint

- American College of Rheumatology (ACR) 20 response at Week 16

Secondary endpoints (at Week 16)

- Change from baseline in the Health Assessment Questionnaire-Disability Index (HAQ-DI) score
- Psoriasis Area and Severity Index (PASI) 75 response
- Change from baseline in the Physical Component Summary (PCS) score of the Short Form-36 (SF-36) Questionnaire

Key additional prespecified endpoints (at Week 16)

- ACR 50 and ACR 70 responses
- Disease Activity Score 28 C-reactive protein: Low disease activity, remission, and change from baseline in score
- Dactylitis: Resolution of dactylitis, change from baseline in Leeds Dactylitis Index (LDI) Basic, change from baseline in dactylitis count
- Enthesitis: Resolution of enthesitis by Leeds Enthesitis Index (LEI) and Spondyloarthritis Research Consortium of Canada (SPARCC) Enthesitis Index, change from baseline in enthesitis count by LEI and SPARCC Index
- Physician's Global Assessment of Fingernails (PGA-F) response of 0/1
- Minimal Disease Activity (MDA) response
- Psoriatic arthritis activity: Psoriatic Arthritis Disease Activity Score (PASDAS) and Disease Activity Index for Psoriatic Arthritis (DAPSA)
- HAQ-DI response (≥0.35 improvement from baseline)

Sample size considerations

A sample size of 60 patients per arm provided 87.5% power to detect a significant dose-response trend compared with placebo for the primary endpoint. The power determination was based on the assumption of a placebo response rate of 30% and deucravacitinib response rates of 50% and 55% in the deucravacitinib 6 mg QD and 12 mg QD groups, respectively, using a linear trend test with $\alpha=0.05$ (1-sided) or $\alpha=0.10$ (2-sided).

Secondary endpoint analyses

Secondary endpoint analyses were conducted sequentially and only proceeded to the next endpoint if the null hypothesis was rejected (α=0.10 [2-sided]) for the prior endpoint demonstrating a positive dose-response for that endpoint. If an endpoint failed at any step, all subsequent *P* values were considered nominal. Strategies for addressing missing data used in the primary efficacy analysis were also used for binary secondary efficacy endpoints. For continuous secondary endpoints, a modified baseline observation carried forward method was used for patients who discontinued study treatment due to lack of efficacy or AEs, or who started a protocol-prohibited treatment. Patients who had discontinued the study treatment for other reasons had missing data imputed using the last observation carried forward method.
