# Supplementary figures and images for: Efficacy and safety of selective TYK2 inhibitor, deucravacitinib, in a phase II trial in psoriatic arthritis
Source: Ann Rheum Dis. 2022 Mar 3;81(6):815–22. doi: 10.1136/annrheumdis-2021-221664 (PMC9120409; doi:10.1136/annrheumdis-2021-221664)

Supplemental Figure S2. Patient Disposition

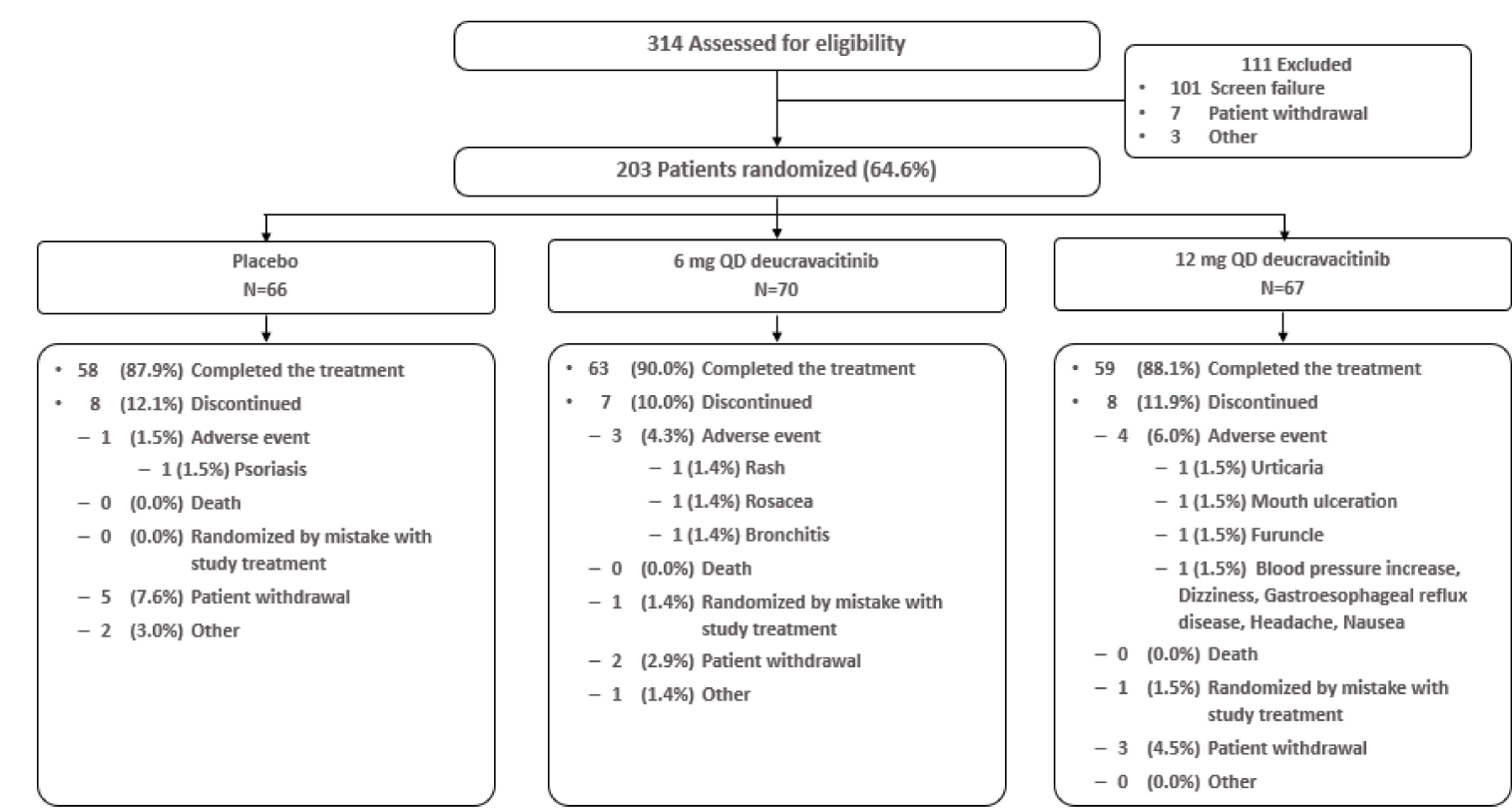

QD, once daily.

Supplement: Supplementary data [file annrheumdis-2021-221664supp003.pdf]
