# Supplementary material for: Efficacy and safety of selective TYK2 inhibitor, deucravacitinib, in a phase II trial in psoriatic arthritis
Source: Ann Rheum Dis. 2022 Mar 3;81(6):815–22. doi: 10.1136/annrheumdis-2021-221664 (PMC9120409; doi:10.1136/annrheumdis-2021-221664)
Supplement: Supplementary data [file annrheumdis-2021-221664supp004.pdf]

Supplemental Figure S3. ACR 20 Response at Week 16 – Effects of TNFi Experience, Body Weight, and Gender

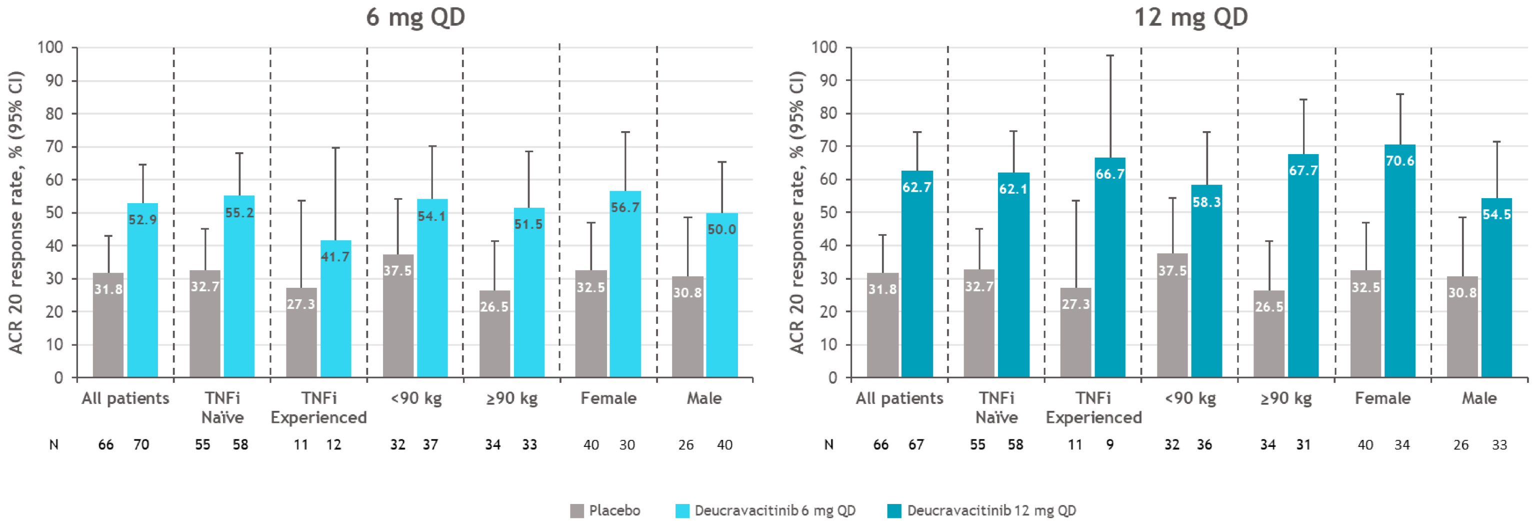

ACR, American College of Rheumatology; CI, confidence interval; QD, once daily; TNFi, tumor necrosis factor inhibitor.
