# Supplementary material for: Efficacy and safety of selective TYK2 inhibitor, deucravacitinib, in a phase II trial in psoriatic arthritis
Source: Ann Rheum Dis. 2022 Mar 3;81(6):815–22. doi: 10.1136/annrheumdis-2021-221664 (PMC9120409; doi:10.1136/annrheumdis-2021-221664)
Supplement: Supplementary data [file annrheumdis-2021-221664supp005.pdf]

Supplemental Table S1. ACR Components at Week 16

		Deucravacitinib	
**Component**	**Placebo n=66**	**6 mg QD n=70**	**12 mg QD n=67**
Tender joint count			
Baseline, mean (SD)	16.9 (9.8)	18.1 (10.3)	19.4 (11.8)
Change from baseline, mean (SD)[a]	-4.6 (9.7)	-9.3 (9.7)	-12.2 (10.2)
% change from baseline, mean (SD)[a]	-25.9 (51.2)	-49.0 (51.9)	-60.4 (35.9)
Swollen joint count			
Baseline, mean (SD)	10.5 (7.7)	11.9 (7.0)	11.3 (9.0)
Change from baseline, mean (SD)[a]	-4.3 (8.0)	-7.7 (5.8)	-8.5 (9.1)
% change from baseline, mean (SD)[a]	-39.8 (63.3)	-68.4 (33.1)	-67.7 (44.1)
Patient Global Assessment of disease activity			
Baseline, mean (SD)	66.2 (15.8)	68.2 (16.8)	63.6 (15.6)
Change from baseline, mean (SD)[a]	-13.4 (23.5)	-28.7 (23.1)	-27.6 (25.8)
% change from baseline, mean (SD)[a]	-18.1 (37.9)	-43.0 (34.0)	-40.4 (49.2)

Patient Global Assessment of pain			
Baseline, mean (SD)	64.9 (18.2)	63.6 (21.7)	63.8 (15.9)
Change from baseline, mean (SD)[a]	-13.8 (21.5)	-25.3 (26.1)	-27.5 (25.0)
% change from baseline, mean (SD)[b]	-20.2 (35.0)	-36.9 (39.1)	-42.0 (37.3)
Physician Global Assessment of disease activity			
Baseline, mean (SD)	63.8 (14.8)	68.2 (14.7)	63.3 (16.1)
Change from baseline, mean (SD)[c]	-19.9 (21.8)	-33.6 (23.0)	-32.2 (25.0)
% change from baseline, mean (SD)[c]	-29.2 (34.7)	-49.1 (32.1)	-47.7 (35.2)
HAQ-DI total score			
Baseline, mean (SD)	1.3 (0.6)	1.3 (0.6)	1.3 (0.6)
Change from baseline, mean (SD)[a]	-0.1 (0.4)	-0.4 (0.5)	-0.4 (0.6)
% change from baseline, mean (SD)[d]	-10.6 (31.2)	-36.0 (39.0)	-30.8 (52.6)
High-sensitivity C-reactive protein			
Baseline, mean (SD)	20.4 (39.1)	17.6 (23.6)	16.5 (21.7)
Change from baseline, mean (SD)[e]	-3.3 (22.6)	-14.2 (24.5)	-10.9 (22.8)
% change from baseline, mean (SD)[e]	36.0 (221.3)	-40.0 (54.0)	-9.1 (144.0)

All calculations were performed on individual patient data. Change from baseline = Week 16 value – baseline value.

% change from baseline = ((week 16 value – baseline value)*100)/baseline value.

ACR, American College of Rheumatology; HAQ-DI, Health Assessment Questionnaire-Disability Index; QD, once daily; SD, standard deviation.

[a]Calculated for patients with valid Week 16 data (placebo, n=58; 6 mg QD, n=63; 12 mg QD, n=60).

[b]Calculated for patients with valid Week 16 data (placebo, n=58; 6 mg QD, n=62; 12 mg QD, n=60).

[c]Calculated for patients with valid Week 16 data (placebo, n=57; 6 mg QD, n=63; 12 mg QD, n=59).

[d]Calculated for patients with valid Week 16 data (placebo, n=58; 6 mg QD, n=62; 12 mg QD, n=59).

[e]Calculated for patients with valid Week 16 data (placebo, n=57; 6 mg QD, n=62; 12 mg QD, n=60).
