# Supplementary material for: Efficacy and safety of selective TYK2 inhibitor, deucravacitinib, in a phase II trial in psoriatic arthritis
Source: Ann Rheum Dis. 2022 Mar 3;81(6):815–22. doi: 10.1136/annrheumdis-2021-221664 (PMC9120409; doi:10.1136/annrheumdis-2021-221664)
Supplement: Supplementary data [file annrheumdis-2021-221664supp008.pdf]

Supplemental Table S4. ACR 20 Response Rate and HAQ-DI Adjusted Mean Change From Baseline Over 16 Weeks

Endpoint	Placebo (N=66)	Deucravacitinib 6 mg QD (N=70)	Deucravacitinib 12 mg QD (N=67)
**ACR 20 response rate[a]**			
Week 1 responders, n/N	2/66	8/70	5/67
Response rate, % (95% CI)	3.0 (0.0, 7.2)	11.4 (4.0, 18.9)	7.5 (1.2, 13.8)
P-value vs placebo		0.0631	0.2657
Week 2 responders, n/N	14/66	11/70	17/67
Response rate, % (95% CI)	21.2 (11.3, 31.1)	15.7 (7.2, 24.2)	25.4 (15.0, 35.8)
P-value vs placebo		0.3889	0.5301
Week 4 responders, n/N	20/66	20/70	20/67
Response rate, % (95% CI)	30.3 (19.2, 41.4)	28.6 (18.0, 39.2)	29.9 (18.9, 40.8)
P-value vs placebo		0.8007	0.9763
Week 8 responders, n/N	21/66	33/70	36/67
Response rate, % (95% CI)	31.8 (20.6, 43.1)	47.1 (35.4, 58.8)	53.7 (41.8, 65.7)
P-value vs placebo		0.0731	0.0108
Week 12 responders, n/N	23/66	43/70	41/67

Response rate, % (95% CI)	34.8 (23.4, 46.3)	61.4 (50.0, 72.8)	61.2 (49.5, 72.9)
P-value vs placebo		0.0021	0.0021
Week 16 responders, n/N	21/66	37/70	42/67
Response rate, % (95% CI)	31.8 (20.6, 43.1)	52.9 (41.2, 64.6)	62.7 (51.1, 74.3)
P-value vs placebo		0.0134	0.0004
HAQ-DI adjusted mean change from baseline[b]			
Week 1, mean (95% CI)	-0.04 (-0.12, 0.05)	-0.12 (-0.21, -0.04)	-0.11 (-0.19, -0.02)
P-value vs placebo		0.0985	0.1747
Week 2, mean (95% CI)	-0.15 (-0.25, -0.05)	-0.17 (-0.27, -0.08)	-0.24 (-0.34, -0.13)
P-value vs placebo		0.6593	0.1657
Week 4, mean (95% CI)	-0.08 (-0.19, 0.02)	-0.22 (-0.32, -0.12)	-0.21 (-0.31, -0.10)
P-value vs placebo		0.0426	0.0677
Week 8, mean (95% CI)	-0.11 (-0.23, 0.02)	-0.30 (-0.43, -0.18)	-0.39 (-0.52, -0.27)
P-value vs placebo		0.0155	0.0005
Week 12, mean (95% CI)	-0.15 (-0.28, -0.02)	-0.37 (-0.49, -0.24)	-0.42 (-0.55, -0.29)
P-value vs placebo		0.0073	0.0010
Week 16, mean (95% CI)	-0.11 (-0.24, 0.02)	-0.37 (-0.50, -0.24)	-0.39 (-0.53, -0.26)
P-value vs placebo		0.0020	0.0008

[a]Missing data were imputed as nonresponders.

[b]Modified baseline observation carried forward was used to handle missing data.

ACR, American College of Rheumatology; CI, confidence interval; HAQ-DI, Health Assessment Questionnaire-Disability Index; QD, daily.
