# Supplementary material for: Efficacy and safety of selective TYK2 inhibitor, deucravacitinib, in a phase II trial in psoriatic arthritis
Source: Ann Rheum Dis. 2022 Mar 3;81(6):815–22. doi: 10.1136/annrheumdis-2021-221664 (PMC9120409; doi:10.1136/annrheumdis-2021-221664)
Supplement: Supplementary data [file annrheumdis-2021-221664supp006.pdf]

Supplemental Table S2. Laboratory Parameters (Mean [SD]) at Baseline and Week 16

Laboratory Parameter	Placebo	Deucravacitinib 6 mg QD	Deucravacitinib 12 mg QD
Lymphocyte counts (10^9/L)			
Baseline	1.8 (0.6)	1.7 (0.6)	1.8 (0.6)
Week 16	1.7 (0.6)	1.8 (0.6)	1.9 (0.6)
Neutrophil counts (10^9/L)			
Baseline	5.1 (2.3)	4.7 (1.6)	5.0 (1.6)
Week 16	4.8 (2.6)	4.1 (1.4)	4.8 (1.5)
Platelet counts (10^9/L)			
Baseline	291.9 (126.8)	269.2 (65.6)	273.1 (80.8)
Week 16	279.0 (101.0)	256.2 (52.4)	256.7 (77.5)
Hemoglobin (g/dL)			
Baseline	13.3 (1.5)	13.9 (1.6)	13.7 (1.5)
Week 16	13.5 (1.3)	14.1 (1.6)	14.0 (1.5)
Total cholesterol (mg/dL)			
Baseline	192.8 (39.6)	193.6 (40.9)	187.3 (32.7)
Week 16	196.6 (40.9)	203.6 (50.6)	194.2 (36.2)
Triglyceride (mg/dL)			
Baseline	134.7 (57.2)	167.3 (226.1)	169.7 (102.2)
Week 16	135.8 (71.1)	163.8 (121.1)	189.5 (127.5)
ALT (U/L)			
Baseline	22.8 (15.2)	24.9 (18.4)	23.9 (12.6)
Week 16	26.6 (22.5)	25.2 (14.0)	27.5 (18.3)
AST (U/L)			
Baseline	18.6 (6.1)	22.4 (12.9)	21.7 (9.0)
Week 16	21.8 (10.3)	22.1 (8.7)	26.6 (32.7)
CPK (U/L)			
Baseline	84.8 (59.0)	106.6 (107.9)	103.4 (89.9)
Week 16	91.1 (51.4)	115.3 (97.6)	488.6 (2785.8)[a]
Creatinine (mg/dL)			
Baseline	0.75 (0.16)	0.82 (0.18)	0.79 (0.17)
Week 16	0.78 (0.17)	0.84 (0.17)	0.80 (0.17)

[a]One patient in the deucravacitinib 12 mg QD group had a CPK increase (21,690 U/L) on Day 113 following a period of intense physical exercise in the previous week. This was considered as a severe AE not related to study drug. Study drug was interrupted due to this event. The patient stopped physical exercise, and the event resolved on Day 119 without treatment.

AE, adverse event; ALT, alanine aminotransferase; AST, aspartate aminotransferase; CPK, creatine phosphokinase; QD, daily; SD, standard deviation.
