# Supplementary material for: Efficacy and safety of selective TYK2 inhibitor, deucravacitinib, in a phase II trial in psoriatic arthritis
Source: Ann Rheum Dis. 2022 Mar 3;81(6):815–22. doi: 10.1136/annrheumdis-2021-221664 (PMC9120409; doi:10.1136/annrheumdis-2021-221664)
Supplement: Supplementary data [file annrheumdis-2021-221664supp007.pdf]

Supplemental Table S3. Shift Table of Maximum Postbaseline Grade (Grades 3 and 4) of Laboratory Parameters

		Maximum Postbaseline Grade, n	
	Baseline Grades (n)	Grade 3	Grade 4
**Lymphocyte count decrease**			
Placebo	Grade 0 (59)	0	0
	Grade 1 (5)	0	0
	Grade 2 (1)	0	0
	Grade 3 (0)	0	0
	Grade 4 (0)	0	0
DEUC 6 mg QD	Grade 0 (67)	1	0
	Grade 1 (2)	0	0
	Grade 2 (1)	0	0
	Grade 3 (0)	0	0
	Grade 4 (0)	0	0
DEUC 12 mg QD	Grade 0 (60)	0	0
	Grade 1 (2)	0	0
	Grade 2 (2)	0	0
	Grade 3 (0)	0	0
	Grade 4 (0)	0	0
**Neutrophil count decrease**			
Placebo	Grade 0 (65)	0	0
	Grade 1 (0)	0	0
	Grade 2 (0)	0	0
	Grade 3 (0)	0	0
	Grade 4 (0)	0	0
DEUC 6 mg QD	Grade 0 (66)	0	0
	Grade 1 (4)	0	0
	Grade 2 (0)	0	0
	Grade 3 (0)	0	0
	Grade 4 (0)	0	0
DEUC 12 mg QD	Grade 0 (63)	0	0
	Grade 1 (1)	0	0
	Grade 2 (0)	0	0
	Grade 3 (0)	0	0
	Grade 4 (0)	0	0
**Platelet count decrease**			
Placebo	Grade 0 (64)	0	0
	Grade 1 (1)	0	0
	Grade 2 (0)	0	0
	Grade 3 (0)	0	0
	Grade 4 (0)	0	0

DEUC 6 mg QD	Grade 0 (69)	0	0
	Grade 1 (1)	0	0
	Grade 2 (0)	0	0
	Grade 3 (0)	0	0
	Grade 4 (0)	0	0
DEUC 12 mg QD	Grade 0 (64)	0	0
	Grade 1 (0)	0	0
	Grade 2 (0)	0	0
	Grade 3 (0)	0	0
	Grade 4 (0)	0	0
**Anemia**			
Placebo	Grade 0 (54)	0	0
	Grade 1 (9)	0	0
	Grade 2 (2)	0	0
	Grade 3 (0)	0	0
	Grade 4 (0)	0	0
DEUC 6 mg QD	Grade 0 (63)	0	0
	Grade 1 (7)	0	0
	Grade 2 (0)	0	0
	Grade 3 (0)	0	0
	Grade 4 (0)	0	0
DEUC 12 mg QD	Grade 0 (56)	0	0
	Grade 1 (7)	0	0
	Grade 2 (1)	0	0
	Grade 3 (0)	0	0
	Grade 4 (0)	0	0
**Cholesterol high**			
Placebo	Grade 0 (35)	0	0
	Grade 1 (30)	0	0
	Grade 2 (0)	0	0
	Grade 3 (0)	0	0
	Grade 4 (0)	0	0
DEUC 6 mg QD	Grade 0 (38)	0	0
	Grade 1 (31)	0	0
	Grade 2 (0)	0	0
	Grade 3 (0)	0	0
	Grade 4 (0)	0	0
DEUC 12 mg QD	Grade 0 (42)	0	0
	Grade 1 (21)	0	0
	Grade 2 (0)	0	0
	Grade 3 (0)	0	0
	Grade 4 (0)	0	0
**Hypertriglyceridemia**			
Placebo	Grade 0 (45)	0	0
	Grade 1 (20)	0	0
	Grade 2 (0)	0	0

	Grade 3 (0)	0	0
	Grade 4 (0)	0	0
DEUC 6 mg QD	Grade 0 (47)	0	0
	Grade 1 (17)	0	1
	Grade 2 (3)	1	0
	Grade 3 (1)	0	0
	Grade 4 (1)	1	0
DEUC 12 mg QD	Grade 0 (35)	0	0
	Grade 1 (21)	1	0
	Grade 2 (6)	2	0
	Grade 3 (1)	1	0
	Grade 4 (0)	0	0
**Alanine aminotransferase increased**			
Placebo	Grade 0 (63)	0	0
	Grade 1 (0)	0	0
	Grade 2 (0)	0	0
	Grade 3 (0)	0	0
	Grade 4 (0)	0	0
DEUC 6 mg QD	Grade 0 (69)	0	0
	Grade 1 (0)	0	0
	Grade 2 (0)	0	0
	Grade 3 (0)	0	0
	Grade 4 (0)	0	0
DEUC 12 mg QD	Grade 0 (64)	0	0
	Grade 1 (0)	0	0
	Grade 2 (0)	0	0
	Grade 3 (0)	0	0
	Grade 4 (0)	0	0
**Aspartate aminotransferase increased**			
Placebo	Grade 0 (65)	0	0
	Grade 1 (0)	0	0
	Grade 2 (0)	0	0
	Grade 3 (0)	0	0
	Grade 4 (0)	0	0
DEUC 6 mg QD	Grade 0 (70)	0	0
	Grade 1 (0)	0	0
	Grade 2 (0)	0	0
	Grade 3 (0)	0	0
	Grade 4 (0)	0	0
DEUC 12 mg QD	Grade 0 (64)	1	0
	Grade 1 (0)	0	0
	Grade 2 (0)	0	0
	Grade 3 (0)	0	0
	Grade 4 (0)	0	0

**Creatine phosphokinase increased**			
Placebo	Grade 0 (63)	1	0
	Grade 1 (2)	0	0
	Grade 2 (0)	0	0
	Grade 3 (0)	0	0
	Grade 4 (0)	0	0
DEUC 6 mg QD	Grade 0 (66)	0	0
	Grade 1 (4)	0	0
	Grade 2 (0)	0	0
	Grade 3 (0)	0	0
	Grade 4 (0)	0	0
DEUC 12 mg QD	Grade 0 (62)	0	1[b]
	Grade 1 (2)	0	0
	Grade 2 (0)	0	0
	Grade 3 (0)	0	0
	Grade 4 (0)	0	0
**Creatinine increased**			
Placebo	Grade 0 (62)	0	0
	Grade 1 (3)	0	0
	Grade 2 (0)	0	0
	Grade 3 (0)	0	0
	Grade 4 (0)	0	0
DEUC 6 mg QD	Grade 0 (64)	0	0
	Grade 1 (6)	0	0
	Grade 2 (0)	0	0
	Grade 3 (0)	0	0
	Grade 4 (0)	0	0
DEUC 12 mg QD	Grade 0 (60)	0	0
	Grade 1 (4)	0	0
	Grade 2 (0)	0	0
	Grade 3 (0)	0	0
	Grade 4 (0)	0	0

[a]Increases from baseline grades are shown in red.

[b]One patient in the DEUC 12 mg QD group had a CPK increase (21,690 U/L) on Day 113 following a period of intense physical exercise in the previous week. This was considered as a severe AE not related to study drug. Study drug was interrupted due to this event. The patient stopped physical exercise, and the event resolved on Day 119 without treatment.

AE, adverse event; CPK, creatine phosphokinase; DEUC, deucravacitinib; QD, daily; SD, standard deviation.
