# Supplementary material for: Efficacy and safety of selective TYK2 inhibitor, deucravacitinib, in a phase II trial in psoriatic arthritis
Source: Ann Rheum Dis. 2022 Mar 3;81(6):815–22. doi: 10.1136/annrheumdis-2021-221664 (PMC9120409; doi:10.1136/annrheumdis-2021-221664)
Supplement: Supplementary data [file annrheumdis-2021-221664supp009.pdf]

Supplemental Table S5. Mean Laboratory Parameter Values Over 16 Weeks

Laboratory Parameter	Placebo	Deucravacitinib 6 mg QD	Deucravacitinib 12 mg QD
**Lymphocytes, 10^9 cells/L**			
Baseline, n	66	70	67
Mean (SD)	1.8 (0.6)	1.7 (0.6)	1.8 (0.6)
Week 1, n	60	70	60
Mean (SD)	1.8 (0.6)	1.8 (0.6)	1.9 (0.6)
Week 2, n	60	62	59
Mean (SD)	1.8 (0.6)	1.8 (0.6)	1.8 (0.7)
Week 4, n	62	65	62
Mean (SD)	1.8 (0.7)	1.8 (0.6)	1.8 (0.6)
Week 8, n	59	61	58
Mean (SD)	1.8 (0.6)	1.8 (0.6)	1.9 (0.7)
Week 12, n	59	63	62
Mean (SD)	2.0 (0.8)	1.9 (0.6)	2.0 (0.6)
Week 16, n	54	61	59
Mean (SD)	1.7 (0.6)	1.8 (0.6)	1.9 (0.6)
**Neutrophils, 10^9 cells/L**			
Baseline, n	66	70	67
Mean (SD)	5.1 (2.3)	4.7 (1.6)	5.0 (1.6)

Week 1, n	60	70	60
Mean (SD)	4.7 (1.9)	4.6 (1.6)	5.3 (1.6)
Week 2, n	60	62	59
Mean (SD)	4.8 (2.3)	4.4 (1.5)	5.2 (1.6)
Week 4, n	62	65	62
Mean (SD)	5.0 (2.2)	4.3 (1.6)	5.3 (2.0)
Week 8, n	59	61	58
Mean (SD)	4.8 (1.8)	4.3 (1.7)	5.0 (1.9)
Week 12, n	59	63	62
Mean (SD)	4.8 (1.8)	4.2 (1.5)	4.8 (1.5)
Week 16, n	54	61	59
Mean (SD)	4.8 (2.6)	4.1 (1.4)	4.8 (1.5)
**Platelets, 10^9 cells/L**			
Baseline, n	66	70	67
Mean (SD)	291.9 (126.8)	269.2 (65.6)	273.1 (80.8)
Week 1, n	62	70	61
Mean (SD)	283.9 (105.2)	279.5 (66.6)	270.0 (77.4)
Week 2, n	62	63	61
Mean (SD)	288.1 (109.4)	275.2 (74.6)	277.9 (93.3)
Week 4, n	62	65	62
Mean (SD)	275.9 (99.4)	270.5 (65.3)	267.1 (86.6)
Week 8, n	60	61	57
Mean (SD)	271.9 (72.1)	261.7 (61.7)	265.5 (77.1)
Week 12, n	58	64	62
Mean (SD)	292.5 (103.4)	255.7 (59.3)	262.4 (75.9)

Week 16, n	53	61	59
Mean (SD)	279.0 (101.0)	256.2 (52.4)	256.7 (77.5)
**Hemoglobin, g/dL**			
Baseline, n	66	70	67
Mean (SD)	13.3 (1.5)	13.9 (1.6)	13.7 (1.5)
Week 1, n	63	70	61
Mean (SD)	13.3 (1.4)	13.7 (1.5)	13.6 (1.4)
Week 2, n	63	63	62
Mean (SD)	13.3 (1.5)	13.7 (1.6)	13.6 (1.5)
Week 4, n	63	65	62
Mean (SD)	13.3 (1.6)	13.8 (1.4)	13.5 (1.5)
Week 8, n	60	61	58
Mean (SD)	13.5 (1.4)	14.1 (1.6)	13.7 (1.4)
Week 12, n	59	64	62
Mean (SD)	13.3 (1.5)	14.0 (1.6)	13.9 (1.4)
Week 16, n	54	61	59
Mean (SD)	13.5 (1.3)	14.1 (1.6)	14.0 (1.5)
**Total Cholesterol, mg/dL**			
Baseline, n	66	70	67
Mean (SD)	192.8 (39.6)	193.6 (40.9)	187.3 (32.7)
Week 4, n	64	66	62
Mean (SD)	188.3 (42.5)	199.7 (44.5)	185.8 (29.2)
Week 16, n	57	61	59
Mean (SD)	196.6 (40.9)	203.6 (50.6)	194.2 (36.2)

Triglycerides, mg/dL			
Baseline, n	66	70	67
Mean (SD)	134.7 (57.2)	167.3 (226.1)	169.7 (102.2)
Week 4, n	64	66	62
Mean (SD)	136.9 (76.7)	157.8 (144.0)	169.3 (106.2)
Week 16, n	57	61	59
Mean (SD)	135.8 (71.1)	163.8 (121.1)	189.5 (127.5)

QD, daily; SD, standard deviation.
